# A comparison of intrathecal magnesium and ketamine in attenuating remifentanil-induced hyperalgesia in rats

**DOI:** 10.1186/s12871-016-0235-9

**Published:** 2016-09-06

**Authors:** Jiehao Sun, Hai Lin, Xiaona Feng, Jiaojiao Dong, Emmanuel Ansong, Xuzhong Xu

**Affiliations:** 1 Department of Anesthesiology, 1st Affiliated Hospital of Wenzhou Medical University, 1# shangcaicun, Wenzhou, 325000 China; 2 Department of Anesthesiology and Pain medicine, 1st Affiliated Hospital of Wenzhou Medical University, Wenzhou, China

**Keywords:** Magnesium, Remifentanil, Hyperalgesia, Ketamine

## Abstract

**Background:**

Activation of NMDA receptors play an important role in the development of remifentanil-induced hyperalgesia. We hypothesized that in addition to ketamine, intrathecal MgSO_4_ could also relieve thermal and mechanical hyperalgesia in rats.

**Methods:**

Initially, 24 Sprague–Dawley rats were divided into control group, remifentanil group, surgical incision group and remifentanil combined with surgical incision group to create an experimental model. Subsequently, 40 rats were divided into control group, model group, model group plus 100 μg MgSO_4_, 300 μg MgSO_4_ and 10 μg ketamine respectively. Paw withdrawal mechanical thresholds and paw withdrawal thermal latency tests were performed at −24 h, 2 h, 6 h, 24 h, 48 h, 72 h and 7 day after the surgical procedure. After behavior assessment on the 7th day, remifentanil was given again to ascertain whether or not NMDA antagonists could suppress the re-exposure of remifentanil-induced hyperalgesia.

**Results:**

Remifentanil administration plus surgical incision induced significant postoperative hyperalgesia, as indicated by decreased paw withdrawal mechanical thresholds and paw withdrawal thermal latency to mechanical and thermal stimulation. In addition to ketamine, intrathecal MgSO_4_ (100, 300 μg) dose-dependently reduced remifentanil-induced mechanical and thermal hyperalgesia. Ketamine had less mechanical hyperalgesia in 6 h (*p* = 0.018), 24 h (*p* = 0.014) and 48 h (*p* = 0.011) than 300 μg MgSO_4_. There was no difference in inhibiting thermal hyperalgesia between the group ketamine and group MgSO_4_ (300 μg). The rats were given remifentanil again 7 days later after the first exposure of remifentanil. The hyperalgesic effect induced by re-exposure of remifentanil was not reversed in any groups of MgSO_4_ or ketamine.

**Conclusions:**

In addition to ketamine, intrathecal administration of MgSO_4_ dose-dependently reduced remifentanil-induced hyperalgesia in a surgical incision mode. Re-exposure to remifentanil 1 week later again produced hyperalgesia, and this was not altered by the prior intrathecal treatments in any 4 groups treated with MgSO_4_ or ketamine.

**Electronic supplementary material:**

The online version of this article (doi:10.1186/s12871-016-0235-9) contains supplementary material, which is available to authorized users.

## Background

Opioids are the standard of care in the treatment of postoperative pain. However, opioids are also associated with the development of paradoxical, pathologic pain that presents as hyperalgesia [1]. Opioid-induced hyperalgesia (OIH) may counteract its own antinociceptive effect, as well as aggravating a pre-existing pain condition after surgery.

Due to its rapid clearance and recovery, remifentanil is frequently used for post-surgical pain and has more predictable therapeutic outcomes [2]. However, hyperalgesia occurs after a brief exposure to remifentanil and contributes to an increase in postoperative pain [3]. Remifentanil can induce latent pain sensitization [4] and can contribute to the transition from acute to chronic pain.

MK-801, a N-methyl-D-aspartate (NMDA) receptor antagonist, showed reversal effect of OIH [4]. Small doses of ketamine also reversed remifentanil induced hyperalgesia (RIH) [5]. However, side effect of ketamine limits its use in clinics. MgSO_4_ also has been proven to antagonize NMDA receptor and hence, it has analgesic effect. MgSO_4_ acts inside the NMDA receptor channel and occludes the Ca^2+^ current in neurons in the dorsal spinal cord, and hence decrease central release of glutamate [6]. Magnesium was reported to not only prevent the delayed and prolonged hyperalgesia [7, 8] but also to enhance the antinociceptive effect [2] of fentanyl administration in rats. Its effect on hyperalgesia could be regarded as similar to ketamine administration.

In the current study, we hypothesized that intrathecal (i.t.) MgSO_4_ could have equal effects in preventing RIH compared with ketamine. We would also test whether or not a single dose of a NMDA receptor antagonist could inhibit re-exposure of remifentanil administration 1 week later after the first remifentanil administration.

## Method

### Animals

The experimental protocol was approved by the Institutional Animal Care Committee, Wenzhou Medical University. Adult male Sprague–Dawley rats (280 ± 330 g) had an acclimation period of at least 1 week. All animals were maintained at a constant temperature (22 ± 1 °C) with 12:12 h light: dark cycle.

### Intrathecal catheter placement

Intrathecal catheters (PE-10 tube, OD: 0.5 mm, ID: 0.25 mm, AniLab Co. Ltd, China) were implanted under sevoflurane anesthesia using the method described by Liu [9] with some modifications. We have not cut the partial process of L3 in order to decrease the injury to rats.

### Surgical incision

Surgical incision was performed under sevoflurane anesthesia using the method described by Brennan [10]. A longitudinal incision was made from the proximal edge of the heel extending toward the toes in the right hind paw. The plantaris muscle was elevated by forceps, leaving the muscle origin and insertion intact. After homostasis, the skin was closed and covered with antiseptic gauze.

To decrease individual error, catheters implantation and surgical incision were performed by Sun and Feng respectively.

Remifentanil was dissolved in normal saline and infused i.v. at a rate of 1.2 μg.kg-1.min-1 for 60 min.

### Pain assay

Mechanical allodynia was evaluated by electronic von Frey anesthesiometer (IITC INC, Life Science instrument, CA, USA). The pressure value was recorded as paw withdrawal mechanical thresholds (PWMT) by transducer (ALMEMO 2450, Ahlborn, Germany). Animals were placed in individual wire cages (20 × 14 × 20 cm) with a mesh bottom (1 × 1 cm) and allowed to acclimatize for 30 min before testing. A 0.8-mm diameter straight filament was used to apply a force to the plantar surface of the right hind paw. Paw withdrawal or licking was considered nociceptive-like responses. The test was repeated 3 times with an interval of 5 min. The mean PWMT was obtained from the average value of the 3 trials.

To evaluate thermal hyperalgesia, paw withdrawal thermal latency (PWTL) was measured by testing equipment (Model 336, Series 8, IITC INC, Life Science instrument, CA, USA). Rats were placed in a clear plastic chamber (22 cm × 12 cm × 12 cm) with a glass floor (5 mm thick) and allowed to acclimatize for 30 min before testing. A radiant heat source was positioned under the glass floor and focused on the plantar surface adjacent to the wound of right hind paw. PWTL was measured by recording the time from the onset of heat stimulus to withdrawal of the hind paw. A cutoff time of 25 s was established to prevent tissue damage. The test was repeated 3 times with an interval of 5 min. The mean PWL was obtained from the average value of the 3 trials.

### Drugs preparation

The following drugs were used: remifentanil hydrochloride (Ultiva) (batch number: 6150406, Ren Fu Co, China), ketamine hydrochloride (1504151, Heng Rui Co, China), Magnesium sulfate (M2643, Sigma. St. Louis, USA), sevoflurane (4Z121, maruishi-pharm.co. Japan).

### Experiment protocol

All of the following experiments were carried out 7 days later after intrathecal catheter implantation.

- Experiment 1
- This experiment comprised four groups of rats (*n* = 6): group C (rats underwent a sham procedure that consisted of the sevoflurane anesthesia and the same volume of saline without incision); group I (rats underwent a surgical incision and the same volume of saline); group R (rats underwent remifentanil infusion without surgical incision); and group RI (rats underwent surgical incision with remifentanil infusion). PWMT and PWTL tests were performed at −24 h, 2 h, 6 h, 24 h, 48 h and 72 h after the surgical incision.
- Experiment 2
- This experiment consisted of five groups of rats (*n* = 8): group C (rats underwent a sham procedure that consisted of the sevoflurane anesthesia and the same volume of saline without incision); group RI (rats underwent surgical incision with remifentanil infusion); group RIK (i.t. 10 μg ketamine was given to the group RI); group RIM_low_ (i.t. 100 μg MgSO_4_ was given to the group RI); group RIM_high_ (i.t. 300 μg MgSO_4_ was given to the group RI). Ketamine, MgSO_4_ and normal saline were injected in a volume of 10 μl using a microinjection syringe 30 min before plantar incision. An additional of 20 μl normal saline was administered to flush the catheter. After the injection, the end of the catheter was plugged. PWMT and PWTL tests were performed at −24 h, 2 h, 6 h, 24 h, 48 h, 72 h and 7 days after the surgical procedure.

On day 7 following behavioral assessments, remifentanil was administered again at a rate of 1.2 μg.kg-1.min-1 for 60 min, similar with the first exposure of remifentanil. Behavior assessment was performed 6 h after the re-exposure of remifentanil.

### Statistical analysis

The primary outcome was the AUC value of mechanical hyperalgesia (0-48 h) after surgical incision and remifentanil infusion. We found that a sample size of 7 rats per group would achieve 80 % power to detect a 20 % reduction in AUC value using one-way ANOVA. Assuming a 10 % drop rate, we recruited 8 rats to each group.

All data are expressed as mean ± SD. Time course data for behavioral tests were performed by repeated measures ANOVA. The AUC value of hyperalgesia were statistically tested by ANOVA test with Bonferroni test as post hoc test. Probability values less than 0.05 were considered significant. Sample size estimates were done using PASS software (PASS 2008, Kaysville, UT, USA). Statistical analyses were done using SPSS 15.0 software (SPSS Inc., Chicago, IL, USA).

## Result

No statistically significant differences were detected between the basal PWMT and PWTL tests of each experimental group (*p* > 0.05).

### RIH model

**Fig. 1:**
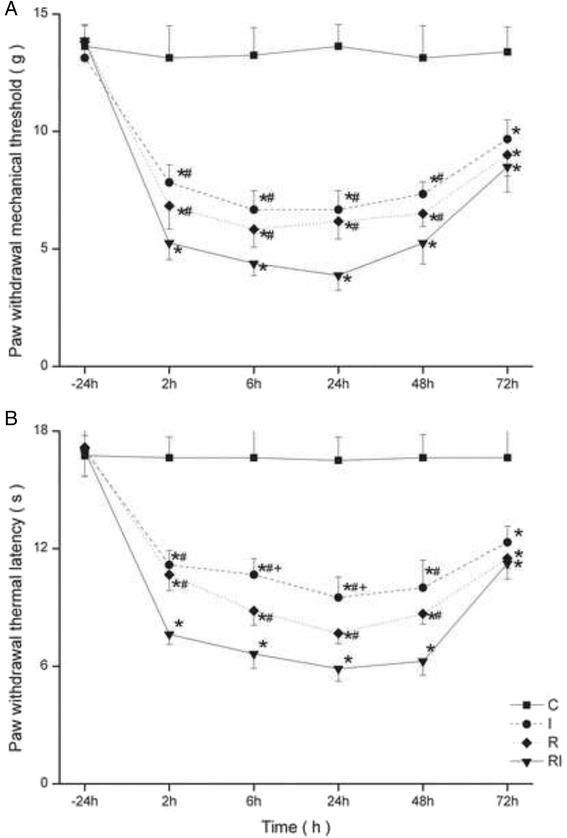
**a**-**b** Paw withdrawal mechanical threshold (**a**) and paw withdrawal thermal latency (**b**) were evaluated at 24 h before incision and 2, 6, 24, 48 and 72 h after remifentanil infusion. Groups allocation: R: Group remifentanil; RI: Group remifentanil + surgical incision; C: Group Control; I: Group surgical incision. Data are expressed as means ± SD. * *P* < 0.001 compared with Group C, # *P* < 0.001 compared with Group RI, + *P* < 0.05 compared with Group R

Repeated measures ANOVA revealed Group RI induced more severe thermal hyperalgesia (F_(3,95)_ =350.641, *p* <0.01) and an interaction between thermal hyperalgesia and time (F_(15,95)_ = 3.041, *p* <0.001). Group RI also had more severe mechanical hyperalgesia (F_(3,95)_ =464.358, *p* <0.01) and an interaction between thermal hyperalgesia and time (F_(15,95)_ = 2.854, *p* <0.001). Group R induced more severe thermal hyperalgesia effect in 6 h (*p* = 0.019) and 24 h (*p* = 0.03) than Group I. (Fig. 1).

### The effect of NMDA antagonists on RIH

**Fig. 2:**
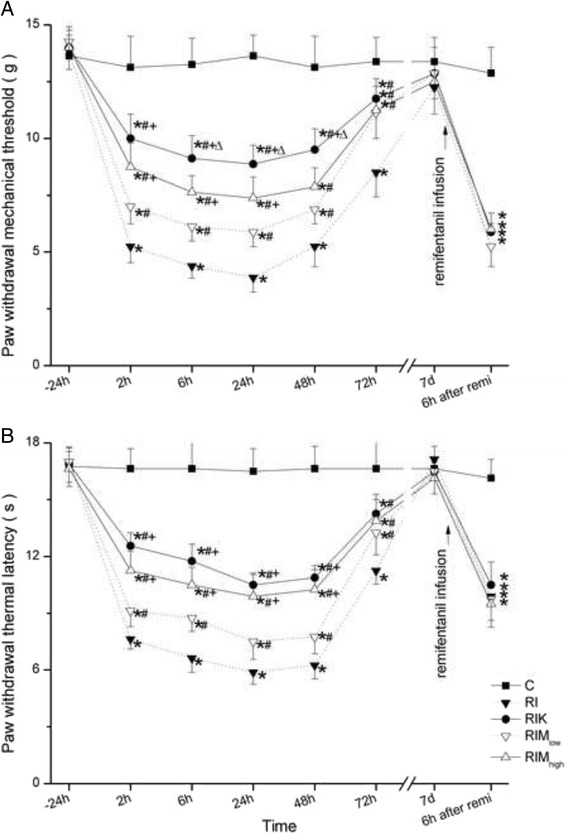
**a**-**b** The NMDA antagonist ketamine and MgSO_4_ were administered intrathecally 30 min before remifentanil infusion and surgical incision. Paw withdrawal mechanical threshold (**a**) and paw withdrawal thermal latency (**b**) were evaluated at 24 h before incision and 2, 6, 24, 48, 72 h, 7 days after remifentanil infusion, 6 h after re-exposure of remifentanil 7 days later. Groups allocation: RI: Group remifentanil + surgical incision; C: Group Control; RIK: Group i.t. 10 μg ketamine was given to the group RI; RIM_low_: Group i.t. 100 μg MgSO_4_ was given to the group RI; RIM_high_: Group i.t. 300 μg MgSO_4_ was given to the group RI_._ Data are expressed as means ± SD. * *P* < 0.001 compared with Group C, # *P* < 0.001 compared with Group RI, + *P* < 0.01 compared with Group RIM_low_, Δ*P* < 0.05 compared with Group RIM_high_

RIH was dose-dependently depressed by MgSO_4_ (100, 300 μg). The treatment with 300 μg MgSO_4_ also significantly suppressed remifentanil-induced thermal hyperalgesia, although it was not statistically different from ketamine treatment. Ketamine had less mechanical hyperalgesia in 6 h (*p* = 0.018), 24 h (*p* = 0.014) and 48 h (*p* = 0.011) than 300 μg MgSO_4_ (Fig. 2).

As shown in Table 1, the AUC for thermal hyperalgesia was lower in the group RIM_low_ compared with the group RI. The AUC for thermal hyperalgesia was lower in group RIK, group RIM_high_ compared with the other three groups at 0–24 h, 0–48 h and 48–72 h (*P* < 0.001). There was no statistical difference in group RIK and group RIM_high_.

**Table 1:** AUC value of NRS scores for thermal and mechanical hyperalgesia

	Group C	Group RI	Group RIM_low_	Group RIM_high_	Group RIK
Thermal hyperalgesia					
AUC_T0-24 h_	431.3 ± 32.4	179.9 ± 12.1*	226.0 ± 11.4*,**	276.5 ± 18.0*, **, ***	301.4 ± 12.5*, **, ***
AUC_T0-48 h_	828.8 ± 60.4	325.4 ± 24.7*	409.0 ± 21.1*, **	518.0 ± 35.8*, **, ***	557.9 ± 18.8*, **, ***
AUC_T48-72 h_	399.0 ± 30.6	210.0 ± 11.1*	252.0 ± 12.8*, **	289.5 ± 18.6*, **, ***	301.5 ± 15.0*, **, ***
Mechanical hyperalgesia					
AUC_M0-24 h_	338.8 ± 27.2	133.5 ± 31.6*	170.9 ± 11.2*	204.6 ± 16.5* **	238.9 ± 25.7*, **, ***
AUC_M0-48 h_	659.8 ± 42.5	243.0 ± 40.2*	323.9 ± 15.6*, **	387.6 ± 25.7*, **, ***	459.4 ± 39.3*, **, ***, ****
AUC_M48-72 h_	318.0 ± 28.7	165.0 ± 20.0*	216.0 ± 12.8*, **	229.5 ± 15.0*, **	255.0 ± 15.4*, **, ***

Data are mean ± SD. *AUC* Area under the curve

Groups allocation: *RI* Group remifentanil + surgical incision, *C* Group Control, *RIK* Group intrathecal 10 μg ketamine, *RIM*_*low*_ Group intrathecal 100 μg MgSO4, *RIM*_*high*_ Group intrathecal 300 μg MgSO4

*: *P* < 0.001 vs group C; **:*P* < 0.001 vs group RI; ***: *P* < 0.01 vs group RIM_low_

****P < 0.01 vs group RIM_high_

As shown in Table 1, the AUC for mechanical hyperalgesia was dose-dependently lower in group RIM_high_ and group RIM_low_ compared with group RI. Group RIK had the lowest AUC of mechanical hyperalgesia. Group RIK had lower AUC of 0 – 48 h compared with group RIM_high_ (*P* = 0.002).

### The re-exposure of remifentanil

Re-exposure to remifentanil resulted in hyperalgesia at 7 days and was not altered by MgSO_4_ or ketamine we gave 7 days before (Fig. 2).

## Discussion

The current study indicated that RIH began from 2nd hour and peaked at 48th hour after remifentanil infusion. Behavioral assessments in current study suggested that thermal hyperalgesia induced by remifentanil was even greater than surgery induced during 24 h after the test, the same with report by Zhang et al. [11].

In order to distinguish RIH from tissue injury of noxious stimulus, we performed the classical rat plantar incision pain model to explore RIH [10]. We selected sevoflurane for anesthesia because it was previously proved to have no influence on nociceptive thresholds [12]. What is the relationship between RIH and opioid receptors for remifentanil? It was reported that blockage of the μ [13], δ [14], or k receptor [4] could precipitates OIH. However, opioids [15] could also cause hyperalgesia in triple knock-out mice which was completely devoid of μ, δ and k opioid receptors [16]. In other words, RIH in rats is not dependent on opioid receptor activity [17].

The mechanism underlying RIH remains controversial. Induced-NMDA current can be potentiated by the application of remifentanil in an in vitro study [18]. By using a two-electrode voltage clamp, remifentanil was found to directly stimulate NMDA receptors of different subunit combinations (NR_1_A/_2_A, NR_1_A/_2_B) in Xenopus laevis oocytes [19]. However, remifentanil itself was not able to stimulate NMDA receptors in spinal cord [20]. Up-regulation of NMDA receptor functions induced by remifentanil may contribute to the clinical development of RIH. It was suggested that enhancing NMDA receptor activity via an intracellular pathway can increase the amount of glutamate [21]. Activation of NMDA receptors could lead to Ca^2+^ entry into the cell, which resulted in a further release of glutamate and more propagation of the pain signal to the brain [20]. NMDA receptor mediated neurotoxicity and apoptosis in the dorsal horn was also involved in RIH after opioid administration [22].

Although MgSO_4_ is weak in antagonizing the NMDA receptor, the results demonstrate that 300 μg MgSO_4_ has equal effect compared with 10 μg ketamine in suppressing thermal hyperalgesia induced by remifentanil administration. For mechanical hyperalgesia, 300 μg MgSO_4_ was not as effective as ketamine.

Mechanical allodynia and thermal hyperalgesia are neurochemically distinct, particularly in relation to the involvement of the NMDA receptor [23]. Thus, NMDA receptor antagonists are more efficacious in reducing mechanical than thermal hyperalgesia in persistent inflammation, and suggesting that mechanical hyperalgesia is mediated through spinal dorsal horn NMDA receptors [24]. In the present study, MgSO_4_ was more effective in inhibiting thermal hyperalgesia, consistent with the results from a previous study [25]. Perhaps NMDA antagonists had a differential susceptibility to express opioid-associated hyperalgesia.

Subcutaneous ketamine did not significantly modify the early analgesic component, but almost completely prevented the delayed decrease in nociceptive threshold after opioid administration [26]. However, the clinical application of ketamine to prevent hyperalgesia is limited because of side effects such as somnolence, dizziness and sedation [27].

Intrathecal administration of MgSO_4_ may be clinically applicable in patients with fentanyl infusion hyperalgesia [28]. Since MgSO_4_ cannot pass through the blood brain barrier due to its high polarity, intrathecal administration can increase Mg^2+^ concentration in the central nervous system with less systemic effect. Intrathecal Mg^2+^ (100 to 500 μg) produced a dose-dependent antinociceptive effect against formalin stimulus [28]. Intrathecal injection of 500 μg MgSO_4_ produced lethargy and slight ataxia, and these symptoms were more pronounced in the rats receiving 750 μg or more [26, 28]. It was reported that 300 μg MgSO_4_ had no motor effects. While intrathecal Mg^2+^ (20 μg) was proved to have no antiallodynic effects [29]. Therefore, the doses of 100 μg, 300 μg and 500 μg of MgSO_4_ were employed in the dose–response analysis in pre-test. 3 of the 4 rats were found to exhibit flaccid paresis in the group which had a dose of 500 μg MgSO_4_ in the pretrial, so we excluded this dose.

It was reported that 10 μg i.t. ketamine intrathecally had no effect on postoperative analgesia [27]. Rota rod test revealed that motor dysfunction was found with the use of i.t. ketamine at doses above 10 μg [27]. So the dose of 10 μg ketamine was selected for use in the current trial.

Some studies have demonstrated that remifentanil can trigger postoperative secondary hyperalgesia [5, 30]. Thus, rats with previous exposure to remifentanil several months earlier exhibit even more severe hyperalgesia after second remifentanil administration [4, 31]. NMDA antagonists was showed to modulate the pre-emptive analgesic efficacy. Epidural infusion of ketamine before transabdominal hysterectomy was reported to reduce pain scores for 2 days after surgery [32]. Pre-emptive treatment with NMDA receptor antagonists cause a lasting change in spinal NMDA receptor complexes which should be more effectively targeted by NMDA receptor antagonists again [33]. In the current trial, We expected to see the anti-nociceptive effect if the intrathecal pretreatment of NMDA antagonists had blocked the hyperalgesia earlier. However, the study found that the NMDA antagonists given 7 days before did not inhibit hyperalgesia induced by re-exposure to remifentanil. Although re-exposure to remifentanil was able to induce hyperalgesia in the present trial, we did not observe that hyperalgesia induced by re-exposure of remifentanil was more severe than the first exposure. It might be attributed to the shorter interval period between the first and second remifentanil administration compared with previous report [4, 31]. In clinics, some operations require multiple sessions. It is common to find patients operated on again 1 week after a first operation. Some patients need to perform emergent traumatic operation first to correct severe physiological status several days before, following by a second operation later. This is the reason the remifentanil re-infusion was made after 7 days in this trial.

### Limitations

1: In this trial, MgSO_4_ was given before remifentanil administration. It has been reported that the ability of MgSO_4_ to suppress hyperalgesia before NMDA activation was lesser from that of the potency after NMDA activation [28]. We did not have the group in which MgSO_4_ was given after remifentanil administration to prove that MgSO_4_ administration before/after remifentanil infusion have different effect of inhibiting RIH. 2: Before we made assessment of re-exposure to remifentanil-induced hyperalgesia, we did not give any additional NMDA receptor antagonist. We postulated that ketamine or MgSO_4_ given 7 days before could still have effect to inhibit RIH but could not be demonstrated in current trial. Further study should aim to investigate whether an intermittent or a continuous exposure to NMDA antagonist will be effective in inhibiting RIH after a second exposure to remifentanil. 3: Many studies have shown that the distribution of NR_2_B subunit in NMDA receptor is limited to the superficial dorsal horn of lumbar spinal cord [11, 14]. The phosphorylation of NR_2_B subunit is related to activation of RIH process. Due to inadequate funding, we did not make western blots to detect the amount of phosphorylation in NR_2_B subunit after MgSO_4_ administration.

## Conclusion

To the best of our knowledge, this is the first demonstration that intrathecal administration of MgSO_4_ and ketamine can suppress RIH. While NMDA receptor antagonists inhibit RIH when given acutely (i.e. on the day of treatment), they do not have a pre-emptive effect to block hyperalgesia resulting from re-exposure to remifentanil 1 week later. Maybe there was no longer any NMDA antagonists available to block the NMDA receptor then. Future studies will need to address the usage or application of MgSO_4_ in a multimodal peri-operative analgesic management targeting the prevention of RIH in clinics.

## Additional file

Additional file 1: Supplementary material. (XLSX 13 kb)

## Abbreviations

NMDA: N-methyl-D-aspartate
NRS: Numeric rating scale
OIH: Opioid-induced hyperalgesia
RIH: Remifentanil-induced hyperalgesia
PWMT: Paw withdrawal mechanical thresholds
PWTL: Paw withdrawal thermal latency
